# A new species of *Paraceratotingis* Henry, Montemayor & Knudson from Guyana (Hemiptera, Heteroptera, Tingidae)

**DOI:** 10.3897/zookeys.796.23076

**Published:** 2018-11-15

**Authors:** Alexander H. Knudson

**Affiliations:** 1 Department of Entomology, North Dakota State University, NDSU Dept. 7650, P.O. Box 6050, Fargo ND 58108-6050, USA North Dakota State University Fargo United States of America

**Keywords:** Heteroptera, lace bugs, *Tigava* complex

## Abstract

*Paraceratotingishenryi***sp. n.**, is described from Guyana. Color photographs and a diagnostic key are provided to aid in distinguishing the species of *Paraceratotingis* Henry, Montemayor, and Knudson. Diagnoses are also provided.

## Introduction

The genus *Paraceratotingis* Henry, Montemayor, and Knudson was erected by Henry et al. (2017) to accommodate two peculiar specimens that were thought to represent a new species of *Ceratotingis*Montemayor (2008). On further examination, Henry et al. (2017) determined that this new species actually belongs in a new genus of the *Tigava* generic complex. Subsequently, while sorting through specimens from the Natural History Museum in London, a single specimen representing another new species of *Paraceratotingis* was discovered, which is described herein.

## Materials and methods

The specimens examined for this paper are from the Natural History Museum (London) (**NHMUK**), and the United States National Museum of Natural History (**USNM**) in Washington, DC. Specimens were examined using a Wild M5 stereomicroscope with 10× eyepieces and an ocular doubler. Precision Digital Positioners (Model 3486-1, Boeckler Instruments, Tucson, Arizona) connected to Microcode Digital Dials (IKL Inc., Newport Beach, California) were used to take measurements, which are given in millimeters (mm). Multiple photographs were taken using a Canon EOS 7D with an Automatic Extension Tube Set (Model DG, Kenko Tokina Co., Ltd., Tokyo, Japan) and a macro photo lens (Model MP-E 65mm, Canon, Inc. Tokyo, Japan) attached to a Stack Shot motorized rail (Cognisys, Inc., Traverse City, Michigan). Photographs were then montaged and edited in Adobe Photoshop CS 6.

## Results

The discovery of a new species of *Paraceratotingis* necessitates a slight modification of the generic description to accommodate the new species. In the original description of the genus, it was stated that the head has three spines, a pair of occipital spines and a median spine. The new species described in this paper lacks the median spine. Additionally, *Paraceratotingis* was originally defined as having the paranota and the costal and subcostal areas of the hemelytra each with two rows of areoles. The new species has all of these areas with only a single row of areoles. The discoidal cell in *P.convergens* Henry, Montemayor, and Knudson (the type species) is closed apically, whereas in the new species the discoidal area is open apically.

### Key to the species of *Paraceratotingis*

**Table:** 

1	Head with pair of occipital spines and median spine; paranota, costal, and subcostal areas of hemelytra biseriate	***Paraceratotingisconvergens* Henry, Montemayor & Knudson**
–	Head with pair of occipital spines, median spine lacking; paranota, costal, and subcostal areas of hemelytra uniseriate	***Paraceratotingishenryi* sp. n.**

#### Paraceratotingis convergens

Taxon classificationAnimaliaHemipteraTingidae

Henry, Montemayor & Knudson, 2017

Paraceratotingis convergens Henry, Montemayor & Knudson, 2017: 272–273, figs 7, 13–14.

##### Diagnosis.

Head with pair of extremely long occipital spines and median spine. Paranota biseriate. Pronotal collar produced upward, forming small, elevated, distinct hood. Hemelytra slightly broader, costal and subcostal areas biseriate; discoidal area closed apically.

##### Material examined.

HOLOTYPE: VENEZUELA: Aragua, El Limón, 4 July 1968, J. Maldonado C. (♂USNM).

#### Paraceratotingis henryi sp. n.

Taxon classificationAnimaliaORDOFAMILIA

http://zoobank.org/A83E8D61-D3AA-4F6F-AE67-E5155BF9230E

Figs 1–3

##### Holotype.

GUYANA: Demerara, Soweyo GDF Camp, 27–30 Sept 1991, JH Martin Coll., BM1991-182 (♀NHMUK). Red handwritten label: Holotype *Paraceratotingishenryi* n. sp. Knudson 2018.

##### Diagnosis.

Head with pair of moderately long occipital spines; medial spine lacking. Paranota uniseriate. Pronotal hood moderately elevated. Hemelytra slightly narrower, costal area uniseriate; subcostal area uniseriate; discoidal area open apically.

**Figures 1–3.**
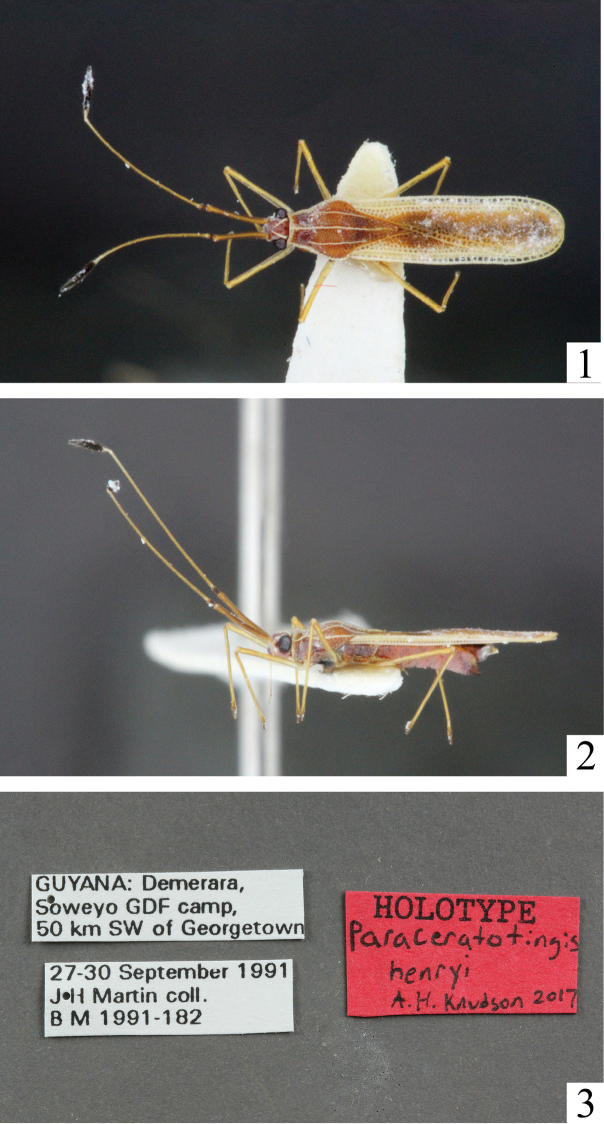
**1** Dorsal habitus of *Paraceratotingishenryi***2** Lateral habitus of *Paraceratotingishenryi***3** Labels of *Paraceratotingishenryi*.

##### Description.

***Head.*** Pale brown; armed with two spines, occipital spines moderately long, converging, but not meeting medially; occipital plates of head lighter in color, obscured by pale wax near bases of antennae; clypeus swollen; eyes bulging, large, 1/3 of head width in dorsal view. Antennae extremely long; segment one long, lightly infuscate, brown, covered with several regular rows of setae; segment two short, concolorous with head, with regular rows of hairs; segment three long, 1.5 times longer than first antennal segment, lighter in color, with regular rows of hairs; segment four clavate, concolorous with preceding segment on basal fourth, black to apex, with longer stouter hairs. Bucculae yellowish, lighter colored than head, bi- to triseriate. Rostrum moderately elongate, apex extended to middle of prothoracic sternite; basal segment brownish, concolorous with basal antennal segment; remaining segments yellow brown except last segment infuscate apically.

***Thorax.*** Pronotum light brown, mostly concolorous with head; tricarinate, punctate, areolate in triangular posterior projection; carinae uniseriate, low, yellowish; pronotal collar lighter in color, slightly tumid, elevated to form hood-like structure; calli large, triangular, lightly pruinose; paranota uniseriate, with minute spinules and pruinescence at outer margins, yellowish. Hemelytra elongate, surpassing abdomen by one fourth to one third its length; outer margin light yellow, with hyaline areolae; costal area of hemelytra uniseriate, with rectangular areolae; subcostal area mostly hyaline, with veins yellowish, subcostal area of wing about two thirds width of costal area, uniseriate with regular areolae; discoidal area poorly differentiated, with five to six rows of areolae at greatest width, open behind; sutural area completely overlapping, wide, with eight to nine rows of areolae at greatest width, cells infuscate, veins brownish,. Hind wings surpassing abdomen in repose, extended halfway between abdomen and hemelytra. Rostral laminae low, uniseriate; sternites dark brown to black. Legs subequal in length, coxae concolorous with pleurites, femora and tibiae elongate, tibiae each with several longitudinal rows of setae, slightly clavate apically, infuscate at apex, with pad of hairs on ventral margin; tarsi darkly infuscate. Ostiolar peritreme small, nearly obsolete.

***Abdomen.*** Light brown, sternites broad; pregenital plate scalloped, with two angular projections along lateral posterior margins; gonocoxae slightly excavated at posterior ventral margin, pruinose.

##### Etymology.

This species is named in honor of Dr. Thomas J. Henry, USDA Systematic Entomology Laboratory, National Museum of Natural History, Washington, DC, for his great contributions to the study of Heteroptera, specifically his advances to the systematics of the Miroidea and Lygaeoidea.

## Discussion

*Paraceratotingishenryi* can be separated from *P.convergens* by the characters provided in the diagnoses. *Paraceratotingisconvergens* is known only from Venezuela, and the new species described herein is known only from Guyana, suggesting that the genus might be endemic to northern South America. No biological information is available for species of *Paraceratotingis*, but a related genus, *Ceratotingis*, has been found feeding and breeding on *Cecropia* sp. (family Urticaceae) in Costa Rica (Kenji Nishida and Paul Hanson, personal communication). *Paraceratotingis* might also feed on members of this genus or other Urticaceae.

## Supplementary Material

XML Treatment for Paraceratotingis convergens

XML Treatment for Paraceratotingis henryi
